# Training the next generation of clinical researchers: evaluation of a graduate podiatrist research internship in rheumatology

**DOI:** 10.1186/1757-1146-6-15

**Published:** 2013-04-16

**Authors:** Serena Naidoo, Catherine Bowen, Nigel Arden, Anthony Redmond

**Affiliations:** 1 Advanced Clinical and Expert Practice, Centre for Innovation and Leadership in Health Sciences, Faculty of Health Sciences, University of Southampton, Southampton SO17 1BJ, UK; 2 Leeds Institute for Molecular Medicine, Division of Rheumatic and Musculoskeletal Disease, University of Leeds, Leeds, UK; 3 NIHR Musculoskeletal Biomedical Research Unit, University of Oxford, Oxford, UK; 4 Department of Rheumatology, Southampton University Hospitals NHS Trust, Southampton, UK; 5 MRC Epidemiology Resource Centre, University of Southampton, Southampton, UK; 6 NIHR Musculoskeletal Biomedical Research Unit, University of Leeds, Leeds, UK

**Keywords:** Podiatry, Rheumatology, Internship, Research, Clinical, Foot, Ankle, Clinical academic career

## Abstract

**Background:**

The aim of this study was to evaluate the effectiveness of the Arthritis Research UK funded graduate internship scheme for podiatrists and to explore the experiences of interns and mentors.

**Methods:**

Nine new graduates completed the internship programme (July 2006–June 2010); six interns and two mentors participated in this study. The study was conducted in three phases. Phase 1: quantitative survey of career and research outcomes for interns. Phase 2 and 3: qualitative asynchronous interviews through email to explore the experiences of interns and mentors. Interpretive phenomenological analysis (IPA) of coded transcripts identified recurring themes.

**Results:**

Research outputs included ten peer reviewed publications with authorial contributions from interns, 23 conference abstract presentations and one subsequent ‘Jewel in the Crown’ award at the British Society for Rheumatology Conference. Career progression includes two National Institute for Health research (NIHR) PhD fellowships, two Arthritis Research UK PhD fellowships, one NIHR Master of Research fellowship and one specialist rheumatology clinical post. Two interns are members of NIHR and professional body committees.

Seven important themes arose from the qualitative phases: perceptions of the internship pre-application; internship values; maximising personal and professional development; psychosocial components of the internship; the role of mentoring and networking; access to research career pathways; perceptions of future developments for the internship programme. The role of mentorship and the peer support network have had benefits that have persisted beyond the formal period of the scheme.

**Conclusions:**

The internship model appears to have been perceived to have been valuable to the interns’ careers and may have contributed significantly to the broader building of capacity in clinical research in foot and ankle rheumatology. We believe the model has potential to be transferable across health disciplines and on national and international scales.

## Background

Evidence based practice represents a key paradigm shift that has taken place in healthcare within the UK over the past decade [1,2]. Being a knowledgeable, aware consumer of research findings is an integral component of modern clinical practice [3]. However, many clinicians lack the time and the research skills to read and interpret the evidence and very few clinicians go on to be full time researchers [4]. More than a decade ago Lenfant [5] predicted a shortage of researchers in the next generation, and indicated that attracting the best minds to biomedical research and retaining them would be a major challenge faced by the research community.

To address difficulties encountered by clinical researchers, particularly those in non-medical disciplines, flexible career pathways for nurse researchers have been proposed [6,7]. Such schemes for allied health professionals (AHPs) are less well developed in the UK however, and for AHPs, direct progression from pre-registration study, through clinical qualification and onto a research career remains uncommon.

One consequence of this research immaturity is the lack of robust evidence in the literature to support even the basic practices in the assessment and management of foot problems associated with rheumatological disease. Despite the increased focus on the assessment and management of musculoskeletal foot and ankle pathology [8-12], systematic reviews continue to report a pressing need for new and better evidence [13-15]. Research in the field of rheumatology and the lower limb has provided insight into the impact of foot problems and evidence for interventions. Podiatry now also has a higher profile within the wider rheumatology community because of this. There is however, a need for building research capacity, developing peer support networks, and a growing need for succession planning.

A funded research internship programme for new graduate podiatrists was developed and ran from July 2006 to June 2010 at the Universities of Southampton and Leeds. The purpose of the internship was to provide early exposure for high achieving young graduates to a professional research culture. Each year, two new graduate clinicians (podiatrists) achieving first class or upper second class honours degree qualifications were recruited through a competitive process coordinated across all twelve schools of podiatry in the UK, to participate in the internship programme. Adverts were sent electronically at the same time to all UK undergraduate podiatry programme leaders, to be cascaded to their final year students and past interns and mentors spoke to students and staff in person. Over five intakes, a total of nine interns each participated in an eight week intensive research placement which introduced them to many diverse aspects of the research process, followed by a two to three year period of mentorship and supported networking. The internship process has been added as Additional file 1.

The aims of this study were to evaluate the effectiveness of this internship model to explore the outputs and career paths resulting from the programme and to explore the interns’ and mentors’ perceptions of their experiences.

## Methods

### Study design

The study utilised a mixed methods approach. A quantitative survey was established to evaluate the internship in terms of output such as post graduate qualifications and research publications were collated empirically. Following this, to further understand the effectiveness of the internship a qualitative interpretative phenomenological analysis, using asynchronous interviews through email, was used to explore the experiences of the clinicians and mentors who participated in the internship programme [16].

### Participants

Nine podiatrists participated in the internship programme between July 2006 and June 2010. Four of the interns completed their undergraduate podiatry degree at the University of Southampton, two came from the University of Brighton and one each from Queen Margaret University in Edinburgh and one from the University of Huddersfield.

The sample investigated was purposive and aimed to include all participants (interns and mentors) who completed the internship programme. Any clinicians who did not complete the internship programme or who did not participate in their internship experience at either the University of Leeds or the University of Southampton were excluded. Additionally, any clinicians/mentors who were unable to complete the email questioning or were unable to give informed consent were excluded.

### Participant recruitment

All clinicians and mentors who completed the internship programme were approached via email invitation by the principal investigator (SN). Potential assenting participants were then recruited individually by email. As suggested by Dillman [17] this aimed to highlight the importance of each individual, encouraging them to participate. Emphasis was placed on maintaining the anonymity and confidentiality of all participants. Approval for this study was obtained from the Faculty of Health Sciences, University of Southampton Research Ethics Committee. All participants gave informed written consent prior to participation.

### Methods of data collection

Demographic data including age at time of internship, gender, qualifications and information regarding research outputs since their involvement in the internship programme (such as poster presentations, journal articles and fellowships) were recorded for each participant. Research outputs were calculated from the time that the internship began, to the time of data collection and therefore do not include outputs from work conducted beyond the period of the internship scheme.

Semi-structured asynchronous interviews were conducted through email to explore the experiences of the clinicians who participated in the internship programme and the experiences of mentors. Online, asynchronous, in-depth interviewing conducted via email is different to an e-mail survey or virtual focus group, as it involves a semi-structured interview conducted between the interviewer and interviewee over an extended period of time [16]. The advantage of e-mail interviewing within this study was that it cost considerably less to administer as potential participants were not co-located but were spread across the UK. Conducting the study via e-mail obviated the need for extensive travel on behalf of either investigator or participants.

All primary questions were directed towards the clinicians’ perceptions of their internship experience and whether this had affected their future career choices. All participants were encouraged to use acronyms, abbreviations and emoticons as well as underlining, capitalisation and the use of exclamation marks for emphasis as a substitute for non-verbal cues. The question lists for both interns and mentors can be accessed as Additional file 2.

### Pilot work/developmental phase of the study

The method of conducting asynchronous interviews was pilot-tested by the primary investigator (SN) prior to the project implementation on one individual who participated in an early version of the internship programme that had run prior to the fully funded scheme.

With e-mail interviewing there is more opportunity for misinterpretation of questions to occur than during face-to-face interviews [16]. The pilot work enabled the primary questions to be tested for validity and any questions that were unclear or were misinterpreted at this stage to be amended to improve clarification. After this phase, the content of the questions were not found to require alteration, however the instructions and structure of the questionnaires were amended.

### Data analysis

The demographic characteristics of the study participants are presented descriptively and the frequency of achievements reported graphically.

Interview transcripts were read and repeat read, in attempt to reduce bias of a single investigator and to gain familiarity with the text [18]. Transcripts were annotated to identify emerging key concepts in an iterative process. Interpretive phenomenological analysis (IPA) of the transcripts was undertaken to identify recurring themes [18,19]. Themes were selected according to the prevalence of descriptions identified in the transcripts, similarities, differences and linguistic connectors in attempt to reduce reflexivity [20,21]. All analyses were conducted by the primary investigator (SN). Extracts from the e-mail interviews have been selected as exemplars to represent the corresponding themes. Each exemplar is identifiable to a unique code to illustrate whether it has come from an intern (IT1-6) or a mentor (MT1-2).

## Results

### Intern participant demographics

From nine clinicians who completed the internship programmes, six participated in this study (1 male, 5 female). The ninth intern (SN) was leading the evaluation and therefore was excluded from participating in this study. Two interns did not respond to the call to participate. Two mentors, one from the University of Leeds and one from the University of Southampton had contributed throughout the four year period of the internships and both participated in the study.

**Figure 1:**
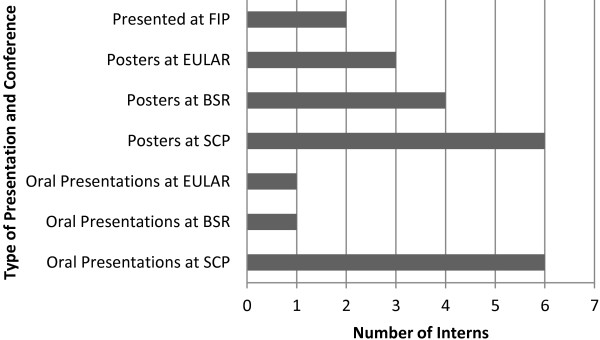
**Research presentation output summary. **Legend: FIP: Federation Internationale des Podologues; EULAR: European League Against Rheumatism; BSR: The British Society for Rheumatology; SCP: The Society of Chiropodists and Podiatrists.

The mean age of the intern participants at the time of commencing their internships was 24 years (range 21–27) and whilst each had completed a BSc (Hons). Podiatry degree, two had also obtained BSc degrees prior to podiatry and one had obtained a diploma. Following the internship each of the intern participants had gone on to present at least one oral or poster presentation at national and European conferences for podiatry and/or rheumatology (Figure 1); totalling 23 conference abstracts. Interns have also, to-date made authorial contributions to ten peer reviewed publications (range 0 to 3) in the podiatry and rheumatology literature [22-31]. One intern had published three papers and another, two papers at the time of data collection. In addition, most of the intern participants reported that they have been successful in obtaining further clinical research training (two NIHR funded PhD fellowships; two Arthritis Research UK funded fellowships and one NIHR MRes Fellowship). Two interns are currently members of NIHR and professional body committees.

The primary aim of the study was to evaluate the effectiveness of the internship programme by exploring the participants’ perceptions of their experiences. The information collected from the email interviews were broken down into seven themes as follows and a sample of representative corresponding quotations are listed in Table 1.

**Table 1:** Quotes to correspond with themes from the analysis


Theme 1	*“I was keen to find out more about the nature of professional research” *IT2
	*“We had an idea for succession planning that would attract bright new podiatry graduates to work with us in our research centres…” *MT1
Theme 2	*“Great opportunity to learn new skills, network, understand what research involves, work with a diverse range of people, work with specialised equipment and further professional development”* IT5
	*“You have to be driven for a career in research. It can be competitive.” *IT5
Theme 3	*“I realised I was capable of greater demands than I originally thought” *IT6
	*“The internship made me excited about podiatry. It opened my eyes to a completely different side of podiatry that I wasn’t exposed to at University” *IT5
Theme 4	*“I think the enthusiasm of the people involved was a key reason for its success” *IT1
	*“They have an energy buzz that surrounds them and a sense of healthy competitiveness that spurs them on to progress and achieve their goals” *MT1
Theme 5	*“An important element has been the pairing of interns across the two institutions and then with previous interns. Peer support seems to be an essential part to the success of the interns’ development, career progression. I doubt that it would work so well if we had taken interns in isolation at each institution.” *MT1
	*“Mentorship was great. You had all levels of support from previous interns, to people from other disciplines” *IT5
Theme 6	*“It has led many onto larger things than I think we could have imagined…I do not think those opportunities for some would have been as easily achieved so early in careers without the internship”* IT6
	*“Being an intern has highlighted just how difficult it is for other podiatrists to be involved in early career research” *IT6
Theme 7	*“Try to establish longer-term programmes to improve continuity of involvement of institutions and consolidations of peer networks”* MT2
	*“I think those who got most out of it before starting PhD were those who came back for research assistant roles and built towards a fellowship. I think it would be great if the internship had provision to help with this kind of role” *IT1

**Legend**: Theme 1: Perceptions of the internship pre-application; Theme 2: Internship Values; Theme 3: Maximising personal and professional development; Theme 4: Psychosocial components of the internship; Theme 5: The role of mentoring and networking; Theme 6: Access to research pathways; Theme 7: Perceptions of future developments for the internship programme.

### Perceptions of the internship pre-application

Each of the interns reported that they had encountered some research exposure prior to commencing the internship but regarded themselves as being unfulfilled and having limited experience.

All interns reported reasons such as a longstanding desire to learn more about professional musculoskeletal research, to conduct research and to further their career, either generically or specifically within their profession.

Two of the interns also reported that they were positively encouraged to apply for the internship by their tutors during the final year of their undergraduate podiatry programme.

From the perspective of the mentors, the application and selection process represented a compromise; “low impact advertising whilst maximising the potential” of attracting the most motivated interns. The programme encountered teething problems in the initial stages due to low exposure amongst podiatry schools which led to difficulties with advertising to and attracting high-achieving graduates, meeting the stringent entry criteria. As the scheme grew in profile and the team gained experience however, these early issues were ameliorated.

### Internship values

The internship was an individual experience for each intern, and no two experiences emerged as being the same. The precise structure of the programme had been tailored to each intern, and therefore the explicit aims and achievements reported by each are diverse. This also links with theme 3: maximising personal and professional development.

Cumulatively, each of the interns reported that they learned specific research skills, the importance of multidisciplinary working and the value of a mutually supportive peer network.

The eight weeks provided a wide range of opportunities and experiences for each intern, providing a chance to improve clinical and research knowledge and skills.

Five of the interns mentioned how this programme allowed them to consider a formal career in research, raising the possibility of different career pathways that had not been previously considered until this programme. This links with theme 6: access to research career pathways.

The mentors discussed how this experience had exceeded their original expectations, creating valuable experiences for all involved and enhancing personal and professional development.

### Maximising personal and professional development

Both personal and professional developments were mentioned consistently by each of the interns in their interviews. Personal confidence was improved and they saw themselves as being more capable than originally envisaged.

Encouragement from international leaders in research and clinical rheumatology and podiatry consolidated this.

A better understanding of higher education, research and clinical rheumatology was also integral in changing the interns’ views of podiatry and their potential for development of extended scope skills to complement their research roles. Their interests in podiatry and rheumatology were stimulated by this programme.

From the mentors’ perspective, they reported finding the internship programme professionally and personally rewarding, as it exceeded expectations, fulfilled their aspirations and created a sense of personal achievement and satisfaction.

### Psychosocial components of the internship

The psychosocial components of the internship differed for each intern and mentor; although recurring enthusiasm, excitement, success, capabilities, inspiration and being privileged were all mentioned.

Competitiveness was recognised by mentors and interns and was interpreted in both a positive and negative light; it was deemed as ‘healthy competitiveness’ from a mentor’s perspective, yet ‘competitive’ from an intern’s viewpoint; for some this created ambition, for others it was difficult to cope with.

### The role of mentoring and networking

The role of mentoring is twofold: peer support and mentor support. The peer support network came into place in 2006 and naturally the most recent interns have been able to benefit most from this system. The on-going mutually supportive network has been able to provide a valuable perspective that can be provided only by someone who has shared the same experiences.

The work across split institutions led one intern to feel that the leadership was disjointed although the others felt mutually supported by both mentors. One mentor felt that this multi-institutional approach was a key element of the programme’s success.

One intern mentioned that the peer support network is still useful on a daily basis now, even though the internship programme has ended. They also felt the support and mentorship has contributed towards defining their career paths.

The opportunity in networking and working arose from a wider range of people than those directly involved in the scheme. Interns noted support from a range of sources including peers and previous interns, plus researchers and clinicians from other disciplines, and reported that this has continued after the internship.

Mentors also felt the peer support was valuable and had supplemented and even exceeded the benefits that had been envisaged originally for the formal mentorship component of the scheme.

By continuing with the programme, consecutive interns have gone on to act as ‘elders’ and therefore mentors themselves and have also aided in the recruitment and selection process of subsequent intakes of interns. Furthermore, the mutually supportive network from both interns and original mentors has encouraged career progression and higher achievement. This links with theme 6: Access to research career pathways.

### Access to research career pathways

The programme allowed interns to recognise that it was possible to have full time careers in research should they choose to do so and then reach the required standards. It highlighted the difficulties for podiatrists in developing an early research career; but accelerated their progression and provided opportunities.

The mentors felt the original aim of attracting high-achieving graduates into rheumatology research and to introduce an alternate career path beyond clinical podiatry has been fulfilled and this is evident by the roles that these podiatrists now have. These roles include clinical posts in rheumatology, competitively funded PhD and MRes fellowships, specialist roles, and research assistant/officers. The internship directly lead to career opportunities for some of the interns, whilst others already had pre-existing employment offers; however in both types of instance the interns reported using the internship offer on their CV or application forms as evidence of personal development beyond their undergraduate programmes of study.

More broadly, the existence and high profile of the internship scheme has led to further interest in clinical research, which has in turn provided more interest in the clinical practice of foot health in rheumatology and in podiatry itself. Encouragement from medical and AHP researchers to continue with further education, especially through prestigious AHP training fellowships, has increased expectations and confidence and led to new career opportunities.

The primary overarching theme emerging from both interns and mentors was importance of the opportunities arising from the programme. Interns felt that access to early research careers have been accelerated, whilst mentors described a golden opportunity for the latest generation of graduates.

### Perceptions of future developments for the internship programme

The majority of interns thought that the programme was too short, and should have continued over a longer time span e.g. 6 months or even 1 year. The mentors agreed.

One of the interns mentioned that a job role commencing after and linked to the programme would maximise the skills learnt. Additionally, mentors identified that being able to link parts of the career pathway would improve long term planning and research capacity development.

The internships were aimed mainly at breadth of research exposure in a short timeframe and so opportunities to see a single project through were limited. It was mentioned that having ownership of a project during the internship provided valuable experience.

In addition, it was thought that increasing the competitiveness in accessing the programme even further by widening the programme to other allied health professionals and nurses would be seen as a positive future development.

## Discussion

We believe we have developed a robust model for internships. The quantitative data indicates that as a result of this internship programme there has been a high level of published output and early engagement with the research process. All of the interns participated in submitting at least one conference abstract in the year of graduating from their podiatry degree. This level of early output exemplifies the opportunities and the career platform provided by participation in the internship. The quality of the outputs was also of a good standard, and while this analysis was not intended to include formal metrics such as citation numbers and H indices, the peer reviewed publications included papers in journals such as Arthritis Care and Research, Rheumatology, BMC Musculoskeletal Disorders and the Journal of Foot and Ankle Research. In addition, outputs have been selected for one Arthritis Research UK Silver Medal award, and a British Health Professionals in Rheumatology Jewel in the Crown award at a national conference, which provides some informal substantiation of the quality of the work being produced by the interns.

In addition to the quantitative data, the use of qualitative methodology to explore both the interns’ and the mentors’ experiences has revealed a number of expectations, feelings, opinions and perceptions associated with participating in the internship and seven key areas of importance have been described. Of particular significance, wanting to advance in a personal and professional capacity, the development and maintenance of mentoring and the peer support network and the psychosocial aspects surrounding the experience were most frequently reported.

Both the mentors and the intern participants had pre-conceptions before entering the internship; the mentors had outlined a personality-type required i.e. high achievers, and very motivated podiatry graduates while the interns were also looking to enhance their careers and achieve great things. Prior to applying, the expectation from both sides was high.

The internships contributed positively to the personal and professional lives of each participant; enhancing self-confidence, maturity and clinical knowledge. Consequently, this has encouraged their involvement in rheumatology foot and ankle research, post-internship. This evaluation indicates that the internships have proved to be valuable on an individual basis and have contributed significantly to the current career choices of each intern, as well as to the broader benefit of building capacity for research in foot and ankle rheumatology.

Lenfant [5] noted the benefits of encouraging broad, multidisciplinary approaches to research at all stages of training; emphasising networking, and collaboration, and linking multiple institutions as being important. Findings from this evaluation support these conclusions and are also consistent with the assertion that mentorship is very important in developing new researchers [5].

Although internships have been proposed more recently in the nursing and midwifery professions as a way of helping unemployed, newly registered professionals to maintain their skills and improve their chances of finding work in Scotland [7], no previous allied health schemes had proposed programmes targeted specifically at potential future researchers. Drotar and colleagues [32] suggest that the challenges of developing the careers of researchers necessitate multifaceted strategies that transcend individual programmes. The narratives within this study have provided unique insights in understanding intern and mentor personal feelings and experiences that exceed their participation in the internship. As such, the findings of this evaluation combined with professional interest are being used to further develop the internship model across other health disciplines both nationally and internationally. For example, in 2011 Arthritis Research UK launched a scheme aimed directly at medical students and the College of Radiographers has also launched a similar scheme.

The interviews were carried out by a previous intern which may have allowed for greater disclosure than if a mentor had been the interviewer. Conversely, using a single interviewer to complete the thematic analysis allowed for potential bias. It is also a limitation of this simple retrospective review that no third party verification of interviews was undertaken. A further technical limitation of e-mail interviewing has been highlighted in the loss of non-verbal cues that may be identified in face-to-face interviews and which can provide additional information. To overcome this to some extent, participants were encouraged to use acronyms, abbreviations and emoticons as well as underlining, capitalisation and the use of exclamation marks for emphasis as a substitute for non-verbal cues. Responses were obtained from only six of the qualifying eight interns, providing slightly less rich data than would be ideal although data saturation was reached. Those who did not complete the internship programme were not included, which precludes any inferences regarding their reasons for not completing and any resulting restricts conclusions to those that relate only to interns who were exposed to the whole scheme. In all cases, the interviews were also carried out upon completion of the whole scheme rather than upon completion of their individual internship experiences. Consequently, the responses given are likely to have been influenced both positively and negatively by subsequent career related activities. A focus of quantity of research outputs has been considered more so than quality. It was not an intention that the internship on its own would provide research outputs of significant impact. However, the aim was to educate and inspire interns to a level at which they could progress into a research career. Therefore, the outputs may not all be directly linked with the internship itself as a stand-alone model, but that they demonstrate fulfilment of the aims of inspiring new graduates to take up careers in podiatry and rheumatology research.

## Conclusion

In summary, this evaluation of the internship programme has identified both interns’ and mentors’ experiences and highlighted areas of benefit and potential areas for improvement. Whilst this was not an aim of the internship, the quantitative data indicate a significant early peak in tangible outputs for young career researchers who would not normally have engaged with the research process for several years, and the qualitative analysis indicates that the scheme provides a tailored and strikingly well regarded platform for bright young graduates to develop and maintain a research career path in rheumatology. This has previously been demonstrated through UK NIHR proposals for clinical academic pathways for nurses, midwives and AHP’s. The role of ‘leader’ mentors was planned into the original programme but the importance of peer mentorship was unexpected and proved extremely important to all participants. Formal entry-level opportunities such as the internship appear to offer a potentially useful model for ensuring a continued flow of bright young graduates into the clinical research career pathway. We believe the model has potential to be transferable across health disciplines and on national and international scales.

## Competing interests

The Arthritis Research UK funded internship programme ran from 2006 to 2010. Funding has now ceased and the scheme has ended. The authors acknowledge the possibility of indirect benefit accruing from the public profile of the scheme. Serena Naidoo was a participant in the internship programme. Catherine Bowen was a grant applicant for the funded scheme and a mentor for the internship programme. Nigel Arden was a grant applicant for the funded scheme and a mentor for the internship programme. Anthony Redmond was a grant applicant for the funded scheme and a mentor for the internship programme.

## Authors’ contributions

CB, NA and AR conceived the study. CB and SN designed the study. SN conducted the email interviews and analysis of data. SN drafted the manuscript with assistance from CB, NA and AR. All authors approved the final manuscript.

## Supplementary Material

Additional file 1 The internship process. Click here for file

Additional file 2 The interview schedule. Click here for file
