# Supplementary material for: Training the next generation of clinical researchers: evaluation of a graduate podiatrist research internship in rheumatology
Source: J Foot Ankle Res. 2013 Apr 16;6:15. doi: 10.1186/1757-1146-6-15 (PMC3637456; doi:10.1186/1757-1146-6-15)
Supplement: Additional file 1 — The internship process. [file 1757-1146-6-15-S1.docx]

**INTERNSHIP PROCESS**

**Application, short listing and interview**.

**Summer internship** (8 weeks)

One week introductory taught component covering aims, learning outcomes, use of reflective log, introduction to research pathway and basic research methods.

Six-week practical internship with exposure to multiple components of the research pathway and hands-on experience.

One week debrief and reflection, presentations of experience to group.

**Ongoing contact**

Supported attendance at conferences of British Society for Rheumatology and Society of Chiropodists ad Podiatrists for up to three years.

Ongoing peer support via Facebook group

Peer and mentor supported development of conference abstracts/ scientific manuscripts as appropriate.

Formal and informal mentorship for job applications and career development.

Assistance with sourcing research roles (eg as research assistants).

Assistance with development of personal fellowship applications.

Reciprocal support and peer mentorship of subsequent cohorts of interns
