# Supplementary material for: Training the next generation of clinical researchers: evaluation of a graduate podiatrist research internship in rheumatology
Source: J Foot Ankle Res. 2013 Apr 16;6:15. doi: 10.1186/1757-1146-6-15 (PMC3637456; doi:10.1186/1757-1146-6-15)
Supplement: Additional file 2 — The interview schedule. [file 1757-1146-6-15-S2.docx]

**Interview Schedule**

| **Questions** | **Prompts** |
| --- | --- |
| 1) Can you tell me why you applied for the internship? | - What motivated you? - When was that? - How did that come about? |
| 2) Where did you do the internship? | - Why did you choose that location? - Were there any professionals of admiration at that specific location that you wanted to work with? |
| 3) What were your original aims and objectives of the internship? | - Did you fulfil these? - How? Or Why not? - How did this make you feel? |
| 4) What did your internship experience involve? | - What were the positive aspects of this? - What were the negative aspects of this? - Did you have any particularly difficult challenges to overcome during this time? - How did you feel about this? |
| 5) Do you think that the internship has benefitted your career? | - How? - Why? Or Why not? - How does this make you feel? - Is success important to you? |
| 6) What would you have done differently given the opportunity? | - How? - Why? |
| 7) What are your plans for the future? | - Is this driven from the initial internship experiences? |
| 8) Do you have any further questions or comments to add ? |  |
